# Muscle Synergy Analysis of Different PAPE Protocols on Side Kick Performance in Elite Sanda Athletes: A Repeated Measures Study

**DOI:** 10.3390/s26010296

**Published:** 2026-01-02

**Authors:** Ziwen Ning, Zihao Chen, Tianfen Zhou

**Affiliations:** School of Wushu, Shanghai University of Sport, Shanghai 200438, China; 2121212012@sus.edu.cn (Z.N.); 2321212020@sus.edu.cn (Z.C.)

**Keywords:** post-activation potentiation, muscle synergy, surface electromyography, sanda athletes, neuromuscular coordination

## Abstract

**Background:** Post-activation potentiation (PAPE) enhances athletic performance through brief, high-intensity reactivation and holds significant application value in competitive sports. As a core offensive and defensive technique in Sanda, the side kick demands exceptional neuromuscular coordination. However, current research on PAPE applications in specialized techniques for competitive sports remains limited. There is a lack of comparative analysis on neuromuscular activation characteristics of the side kick in high-level Sanda athletes across different PAPE protocols, and the optimal adaptation scheme remains unidentified. Muscle coordination analysis based on non-negative matrix factorization (NMF) offers an objective perspective to elucidate the neuromuscular control mechanisms underlying this technique, thereby addressing this research gap. **Methods:** Eighteen high-level Sanda athletes (National Level 1 or above) participated in a randomized crossover design, sequentially undergoing three PAPE protocols—ESG, RBG, and SQG—with 10-day intervals between each intervention. Using the Noraxon wireless surface electromyography system, high-speed cameras, and the MY JUMP APP, we simultaneously collected vertical jump height data at different time points (6, 8, 10 min) post-intervention, along with electromyography and kinematic data of the side kick movement 6 min post-intervention. The NMF algorithm was employed to extract muscle coordination features (activation weights, activation coefficients), and repeated measures ANOVA or Friedman tests were used to assess intergroup differences. **Results:** Vertical jump height was significantly higher in the ESG group than in the RBG group at 6, 8, and 10 min post-intervention (*p* < 0.05). At 6 min post-intervention, it was also significantly higher than in the SQG group (*p* < 0.05). SQG showed significantly higher ESG than RBG at 8 min post-intervention (*p* < 0.05), with no significant differences from the other two groups at 10 min. Regarding muscle coordination, ESG and SQG exhibited significantly higher right rectus femoris activation weights than RBG (*p* < 0.05); ESG’s gluteus maximus and rectus femoris activation weights were significantly higher than RBG (*p* < 0.05), with generally longer activation durations across all synergistic modules compared to the other two groups. Although RBG’s vastus lateralis and gluteus medius activation weights were significantly higher than some groups, this did not translate into overall performance advantages. **Conclusions:** Different PAPE protocols exert distinct effects on vertical jump height and muscle coordination patterns during side kicks in elite Sanda athletes. The combined electrical stimulation protocol, which combines the immediate and sustained effects of PAPE, effectively enhances key muscle activation weights and prolongs coordination module activation duration. It represents the optimal solution for optimizing neuromuscular activation characteristics during sidekicks.

## 1. Introduction

Post-activation potentiation (PAPE) is a physiological mechanism that can acutely enhance motor performance by activating neuromuscular regulatory pathways through prior high-intensity, short-duration muscle contractions [1,2,3]. While long-term systematic training remains the fundamental basis for athletic development, PAPE offers a complementary strategy to optimize performance in trained individuals. Its potential value lies in the ability to temporarily elevate muscle contraction efficiency, augment central nervous system drive, and improve inter-muscular coordination through precise pre-activity protocols. This may reduce movement economy and support the precise expression of force during explosive, skill-based actions [4]. Research indicates that PAPE can enhance power output and movement stability in sports such as basketball and swimming. When integrated into a periodized training plan, it may serve as a useful tool for pre-competition priming and sport-specific skill potentiation in elite athletes, potentially helping to fine-tune peak performance [5,6].

Sanda, also known as Chinese Combat, is a modern full-contact competitive combat sport originating from Chinese martial arts. Its technical system highly integrates punching, kicking, and grappling techniques, emphasizing the three-dimensional tactical principle of “kick from a distance, strike up close, and grapple at close quarters”. It places extremely high demands on athletes’ strength, speed, neuromuscular coordination, and tactical adaptability. During competition, athletes engage in full-contact sparring on a padded ring, aiming to score points by landing effective strikes or executing takedowns. In this study, the side kick emerges as a core and critical technique within Sanda [7]. As a core offensive and defensive technique in Sanda, the side kick combines long attack range, explosive power, and broad control coverage. It serves as a critical means for scoring, counter-defense, and spatial control during combat, with its technical quality directly influencing match outcomes [8]. This movement requires a continuous biomechanical process of “push-off, rotation, extension, and force generation”, involving the coordinated participation of multiple muscle groups including lower-body push-off muscles, core stabilizers, and trunk regulators. It demands exceptional neuromuscular synchronization, force transmission efficiency, and movement precision [9]. For elite Sanda athletes whose techniques have reached maturity, traditional training offers limited potential for performance enhancement [10]. The core challenge in specialized training lies in scientifically optimizing the neuromuscular activation patterns of the side kick, improving its movement economy, and enhancing its practical applicability in combat.

Surface electromyography (sEMG), as a non-invasive, high-temporal-resolution biosignal acquisition technique, has been widely applied in the fields of exercise science, rehabilitation medicine, and sports engineering. By recording the electrophysiological signals generated by muscle activity, electromyography sensors objectively reflect the activation state, temporal characteristics, and coordination patterns of the neuromuscular system during movement, providing quantitative evidence for motion analysis, training optimization, and injury prevention. In recent years, advancements in wireless transmission, multi-channel synchronous acquisition, and signal processing algorithms have deepened the application of sEMG sensors in competitive sports, demonstrating significant value particularly in sport-specific technique analysis, fatigue monitoring, and performance evaluation. In combat sports such as Sanda, boxing, and judo, sEMG technology has been employed to analyze force characteristics, muscle coordination patterns, and bilateral coordination mechanisms during key technical movements, providing critical data support for scientific training [11].

The emergence of advanced wearable sensing technologies has revolutionized the collection and analysis of biomechanical and physiological data in sports science, providing a bridge between laboratory-grade measurements and real-world athletic environments. In particular, lightweight, wireless surface electromyography (sEMG) systems, such as the one utilized in this study, exemplify this wearable paradigm. They enable the non-invasive, high-fidelity capture of neuromuscular activity during dynamic, sport-specific movements with minimal movement restriction [12]. This study leverages such wearable sEMG technology to investigate the acute effects of PAPE on muscle synergy patterns during the Sanda side kick. By employing a multi-channel, wireless setup, we move beyond traditional performance metrics (e.g., jump height) to obtain a portable, detailed, and objective mapping of the neuromuscular coordination strategies underpinning technical performance. Therefore, this work not only addresses the effects of different PAPE protocols but also demonstrates the practical application of wearable sensors in delivering nuanced, athlete-specific neurophysiological insights, contributing to the field of precision training in combat sports.

Non-negative Matrix Factorization (NMF) serves as a core method for analyzing muscle coordination patterns. It extracts physiologically meaningful muscle coordination structures from complex electromyographic signals, quantifies the central nervous system’s integrated regulatory strategies for multiple muscles, and provides an objective, precise analytical perspective for revealing the neuromuscular control mechanisms underlying athletic techniques [13]. Current PAPE research predominantly focuses on enhancing fundamental strength performance [14], with limited application studies targeting specialized techniques in combat sports like Sanda. Particularly lacking are comparative analyses of neuromuscular activation characteristics in high-level athletes performing sidekicks under different PAPE protocols, leaving unclear which PAPE intervention optimally aligns with the technical demands of the side kick.

Therefore, this study aimed to identify the optimal post-activation potentiation (PAPE) protocol for enhancing the neuromuscular performance of the side kick in elite Sanda athletes. To achieve this, we employed a randomized crossover design with high-level athletes and utilized non-negative matrix factorization (NMF) to analyze and compare the muscle synergy patterns, neural drive efficiency, and activation timing of the side kick following three distinct PAPE interventions. The findings are intended to provide scientific evidence and practical guidance for the precision training and personalization of this essential technique.

## 2. Research Subjects and Methods

### 2.1. Research Subjects

This study employed G*Power 3.1 software for pre-test sample size calculation. Repeated measures ANOVA was selected as the statistical method, with effect size f = 0.30, α = 0.05, statistical power (1–β) = 0.80, three intra-group measurements, and a correlation coefficient of 0.5. Calculations indicated a minimum sample size of 16 participants to achieve adequate statistical power. The study ultimately included 18 high-level Sanda athletes, meeting the sample size requirement. Recruited 18 national-level or higher sanda athletes with five or more years of training experience. All athletes were sourced from provincial or municipal professional teams, with an average training duration of 7.8 ± 2.5 years, age of 22.5 ± 3.1 years, height of 176.5 ± 6.2 cm, and weight of 70.2 ± 18.3 kg. Participants had no history of neurological disorders or significant lower limb injuries and signed informed consent forms prior to the experiment. Basic information is presented in Table 1. This research protocol was reviewed and approved by the Ethics Committee for Sports Science at Shanghai University of Sport. All participants received detailed explanations of the study content and potential risks prior to the experiment and signed written informed consent forms. The experimental process strictly adhered to the ethical guidelines of the Declaration of Helsinki, ensuring the rights and safety of participants.

### 2.2. Research Methods

#### 2.2.1. Experimental Equipment

Surface Electromyography Acquisition System: Utilizes the Noraxon wireless surface sEMG system (Noraxon U.S.A. Inc., Scottsdale, AZ, USA), which operated at a sampling frequency of 2000 Hz with a signal bandwidth 10–500 Hz, common-mode rejection ratio > 110 dB, compliant with SENIAM standards.

High-Speed Camera: Utilizes a 200 Hz model. Prior to experiments, three-dimensional spatial calibration is performed using a 12-point calibration frame (1 × 1 × 0.8 m), with a reprojection error < 0.3 mm.

Vertical Jump Height Testing Device: Utilized the MY JUMP APP (iPhone version) to capture vertical jump height. Vertical Jump Height Testing Device: The My Jump 2 application (Version 2.0.1 for iOS) was used to capture vertical jump height. This application analyzes flight time from takeoff to landing using the smartphone’s high-speed camera function and calculates height based on physics formulas. Its validity and reliability have been extensively validated [15]. The vertical jump test was employed as a well-validated, non-invasive proxy for assessing lower-limb explosive power and neuromuscular readiness. While it does not directly measure the force or velocity of the side kick, improvements in vertical jump height following PAPE interventions are indicative of an enhanced state of the neuromuscular system’s capacity for rapid force production, which is a fundamental physical quality underpinning the performance of explosive technical movements like the side kick [16].

Intervention Equipment: Eleiko barbells were used for squat training. Umay resistance bands provided variable resistance. Neuromuscular electrical stimulation (NMES) employed the Compex SP 8.0 stimulator (biphasic square wave, 75 Hz, pulse width 400 μs, intensity at 90% of maximum tolerated level). Standard Sanda protective gear and punching bags were uniformly used during testing to ensure equipment consistency.

#### 2.2.2. Test Actions

Maximum Voluntary Contraction (MVC) Test: Conducted prior to formal testing, each target muscle performs 3 maximum voluntary contractions, with the maximum value used for electromyographic signal normalization.

Side Kick Test: Perform 6 sidekicks at maximum speed against a punching bag on a standard Sanda ring. Target the punching bag at the subject’s chest/rib height. sEMG and kinematic data.

Vertical Jump Height Test: Vertical jump height is measured using the MY JUMP APP (iPhone version). At each designated testing time point (6, 8, and 10 min post-intervention), subjects perform 3 arm-swing-free vertical jumps. The highest value is recorded for subsequent analysis.

#### 2.2.3. Intervention Methods and Intensity

A randomized crossover design was employed, with all subjects sequentially receiving three interventions at 10-day intervals, preceded by a 15 min warm-up session. The specific protocol is detailed in Table 2.

#### 2.2.4. Testing Muscle Selection

Based on the biomechanical characteristics of the side kick movement and prior research foundations, this study selected 15 muscles closely associated with the movement for sEMG signal acquisition. The selected muscles included: brachioradialis (BR), biceps brachii (BB), triceps brachii (TB), anterior deltoid (AD), external oblique (EO), gluteus maximus (GM), gluteus medius (GMed), biceps femoris (BF), rectus femoris (RF), vastus lateralis (VL), Tibialis Anterior (TA), Gastrocnemius Medial Head (GAS), as well as the right Tibialis Anterior (TAR), right Gluteus Maximus (GMR), and right Rectus Femoris (RFR). The aforementioned muscles encompass the primary agonists and synergists involved in key movement components during the side kick: trunk stabilization, hip flexion and extension, knee extension, and ankle flexion/extension. This selection comprehensively reflects the neuromuscular activation pattern and bilateral coordination characteristics of this technical movement [17].

### 2.3. Data Acquisition

#### 2.3.1. Vertical Jump Height Acquisition

To accurately measure subjects’ vertical jump height, this study employed the smartphone application My Jump App for data collection. The measurement procedure is as follows:

(1) Equipment Setup: Secure the smartphone on a tripod with the camera positioned horizontally and approximately 2–3 m from the subject’s jump area, ensuring full-body movement is clearly visible.

(2) Jump Execution: Subjects stand within the measurement area and perform vertical jumps without arm swing. Each jump is spaced at least 30 s apart to prevent fatigue.

(3) Video Recording: Use the My Jump App to record the entire process from takeoff to landing, ensuring no significant obstructions appear in the footage.

(4) Height calculation: The application automatically identifies the start and end frames of the jump and calculates the jump height based on airtime (formula:$\;h=\frac{1}{8}gt^{2}$, Among them: g = 9.81 m/s^2^.

(5) Data recording: At each designated test time point (6, 8, and 10 min post-intervention), subjects completed three vertical jumps, with the highest value recorded for subsequent analysis.

#### 2.3.2. Electromyography Data Acquisition

**Figure 1:**
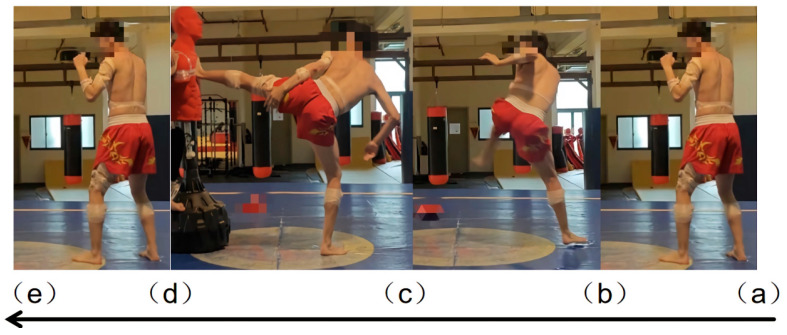
Phases of the Side Kick Movement: preparation phase (**a**,**b**), knee lift phase (**b**,**c**), kicking phase (**c**,**d**), and recovery phase (**d**,**e**).

sEMG signals and kinematic data from 15 target muscles were simultaneously acquired using the Noraxon wireless sEMG system and a high-speed camera. To ensure consistency and reproducibility, the placement of all sEMG electrodes was conducted in strict accordance with a detailed, pre-defined experimental protocol. This protocol integrated the SENIAM recommendations for applicable lower limb muscles and, for muscles not covered by SENIAM (e.g., brachioradialis, biceps brachii, triceps brachii, anterior deltoid, external oblique), was based on well-established electrode placement protocols derived from previous research on similar dynamic movements [18]. The side kick motion was divided into four consecutive phases based on biomechanical characteristics: preparation phase (a,b), knee lift phase (b,c), kicking phase (c,d), and recovery phase (d,e) (Figure 1).

### 2.4. Data Processing

Muscle synergies, intermuscular coherence, and vertical jump height analyses were performed using SPSS statistical software, R software (version 4.2.0), the Muscle Synergies v1.2.5 package, and custom Python(version 3.12) scripts. The specific workflow is as follows:

#### 2.4.1. Data Extraction and Preprocessing

Regarding the number of movement cycles used for synergy extraction, it is acknowledged that studies investigating highly stereotypical, repetitive cyclic movements (e.g., walking or cycling) often recommend analyzing a larger number of cycles (e.g., 20–40) to achieve a robust estimation of muscle synergy structures [19,20]. However, the present study focused on a discrete, maximal-effort, sport-specific skill—the Sanda side kick. For such explosive, high-intensity technical movements, the primary methodological consideration is to balance data representativeness against the confounding influence of fatigue, which itself can significantly alter neuromuscular coordination patterns if too many consecutive trials are performed. Consequently, our protocol of six trials per condition was designed to capture consistent, peak-performance attempts while minimizing fatigue. This approach is consistent with established methodologies in sports science research analyzing single, skilled motor tasks. For instance, a recent study investigating muscle activation during the badminton jump smash—a similarly discrete, explosive skill—successfully extracted and compared muscle synergy characteristics based on the analysis of a single representative movement cycle [21]. Therefore, our use of six averaged trials provides a more substantial data foundation than single-cycle analyses while remaining appropriate for the physiological and technical constraints of maximal sporting actions. The high and consistent variance accounted for (VAF > 90%) across participants and conditions further supports the reliability of the synergy patterns derived from this number of repetitions for the task under investigation.

Using synchronized data from a high-speed camera and a sampling frequency electromyography system, the complete cycle of each side kick movement (from preparation to end of recovery) was defined based on kinematic characteristics, and high-frequency electromyography data corresponding to this period was extracted. A 4th-order Butterworth bandpass filter (20–400 Hz) was applied to remove artifacts. Following full-wave rectification, signals were smoothed through a 4th-order 20 Hz low-pass filter. Data were normalized based on each subject’s maximum sEMG value, and timelines were interpolated to 100 data points to eliminate cycle duration variations.

For each participant and under each experimental condition (ESG, RBG, SQG), the preprocessed sEMG data from the 6 repeated side kick trials were first time-normalized individually (by interpolating each trial’s data to 100 points across the movement cycle). Subsequently, the time-normalized data from all 6 trials were averaged to create a single, representative sEMG profile for that condition. This averaged profile, encompassing the 15 muscles, was then used as the input matrix for the subsequent Non-negative Matrix Factorization (NMF) analysis. This approach of using averaged data enhances the signal-to-noise ratio and provides a more robust estimate of the underlying muscle synergy patterns for each condition.

#### 2.4.2. Muscle Synergy Extraction

The non-negative matrix factorization (NMF) algorithm was employed to extract muscle synergy features using a custom Python script based on the scikit-learn library (version 1.1.3). The non-negative matrix factorization (NMF) algorithm is employed to extract muscle synergy features. The muscle activity matrix D(t) is decomposed into time-invariant synergy vectors W_i_ (muscle weights) and time-varying activation coefficients C_i_(t), with the reconstruction formula defined as:

$$D(t)=\sum_{i=1}^{N_{\mathrm{syn}}}C_{i}{(t)W}_{i}$$

The term $N_{\mathrm{syn}}$ denotes the number of synergies required to explain at least 90% of the variance in electromyographic reconstruction (VAF). The VAF is calculated as follows:

$$\mathrm{VAF}=1\;-\;\frac{\mathrm{SSE}}{\mathrm{SST}}$$

SSE denotes the sum of squared errors, while SST represents the total sum of squares.

#### 2.4.3. Vertical Jump Height Analysis

The application automatically identifies the start and end frames of a jump, calculating jump height based on airtime (formula: $h=\frac{1}{8}gt^{2}$, g = 9.81 m/s^2^. This method has been validated by multiple studies for its high reliability and validity [7], making it suitable for non-invasive, convenient assessment of vertical jump performance.

#### 2.4.4. Statistical Analysis

Statistical analysis was performed using SPSS 26.0 software. The primary outcome measures for this study were: (1) vertical jump height (cm) at 6, 8, and 10 min post-intervention, and (2) muscle synergy characteristics extracted via NMF, including muscle activation weights and activation coefficients (specifically activation duration T). For normally distributed data (assessed using the Shapiro–Wilk test), a two-way repeated measures analysis of variance (RM-ANOVA) was conducted to examine the main effects and interactions. The analysis for vertical jump height included two within-subject factors: ‘Intervention Protocol’ (3 levels: ESG, RBG, SQG) and ‘Time’ (3 levels: 6, 8, 10 min post-intervention). The analysis for muscle synergy parameters (activation weights and coefficients) included one within-subject factor: ‘Intervention Protocol’ (3 levels: ESG, RBG, SQG). In cases of significant main effects or interactions, post hoc pairwise comparisons were performed using paired t-tests with Bonferroni correction. Partial eta squared (ηp^2^) was calculated and reported as the measure of effect size for ANOVA results, with values interpreted as small (0.01), medium (0.06), and large (0.14). For data that violated the assumption of normality, the non-parametric equivalent, Friedman’s test, was used. Post hoc analyses for Friedman’s test were conducted using Wilcoxon signed-rank tests with Holm correction. For these non-parametric comparisons, Cohen’s d was calculated based on the Z-score and sample size to indicate the standardized effect size. The significance level (α) was set at *p* < 0.05 for all statistical tests. Data are presented as mean ± standard deviation (SD) in the text and tables.

## 3. Results

### 3.1. Vertical Jump Height

**Figure 2:**
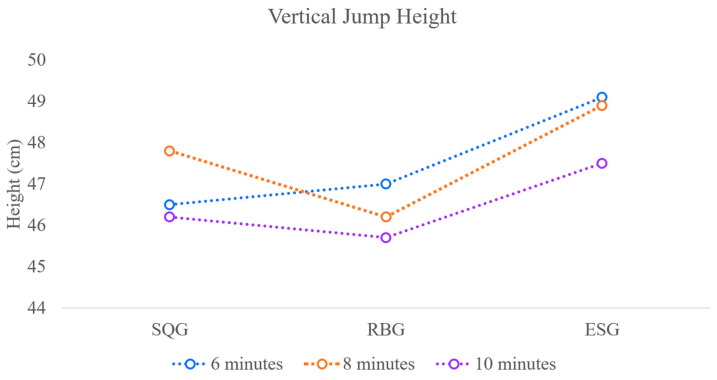
Visualization of Vertical Jump Height Across Groups at Different Time Points Following Three Intervention Measures.

The vertical jump height test results for athletes in each group at specified time points following three different post-activation potentiation interventions are shown in Table 3 and Figure 2. The results of the two-way repeated measures ANOVA (Intervention Protocol × Time) for vertical jump height revealed a significant main effect of Intervention Protocol (F(2, 34) = 7.42, *p* = 0.002, ηp^2^ = 0.30), a significant main effect of Time (F(2, 34) = 5.18, *p* = 0.011, ηp^2^ = 0.23), and no significant Intervention Protocol × Time interaction (F(4, 68) = 0.89, *p* = 0.472, ηp^2^ = 0.05).

At 6 min post-intervention, the ESG group exhibited significantly higher vertical jump heights than both the SQG and RBG groups (*p* < 0.05), while no significant difference was observed between the latter two groups.

By 8 min post-intervention, SQG reached peak vertical jump height, significantly exceeding RBG (*p* < 0.05). ESG remained significantly higher than RBG (*p* < 0.05), but showed no statistically significant difference compared to SQG.

At 10 min post-intervention, only the vertical jump height of the ESG group remained significantly higher than that of the RBG group (*p* < 0.05). No significant differences were observed between the SQG and RBG groups, nor between the SQG and ESG groups.

### 3.2. Muscle Synergy

#### 3.2.1. Muscle Activation Weights

**Figure 3:**
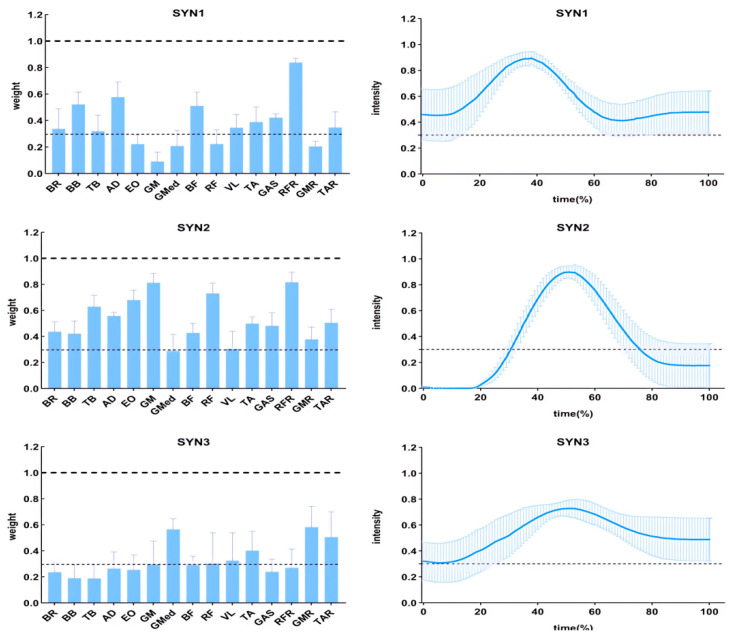
Muscle weights (**left**) and activation curves (**right**) for each synergistic muscle group in ESG.

**Figure 4:**
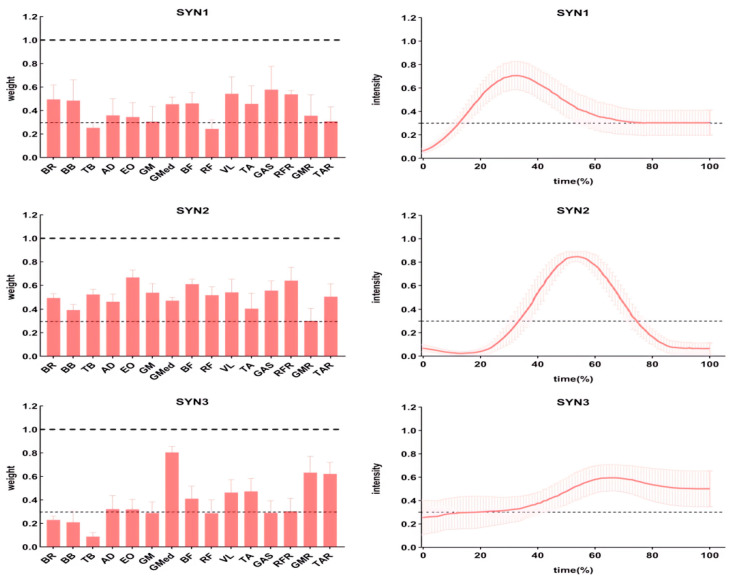
Muscle weights (**left**) and activation curves (**right**) for each synergistic muscle group of the RBG.

**Figure 5:**
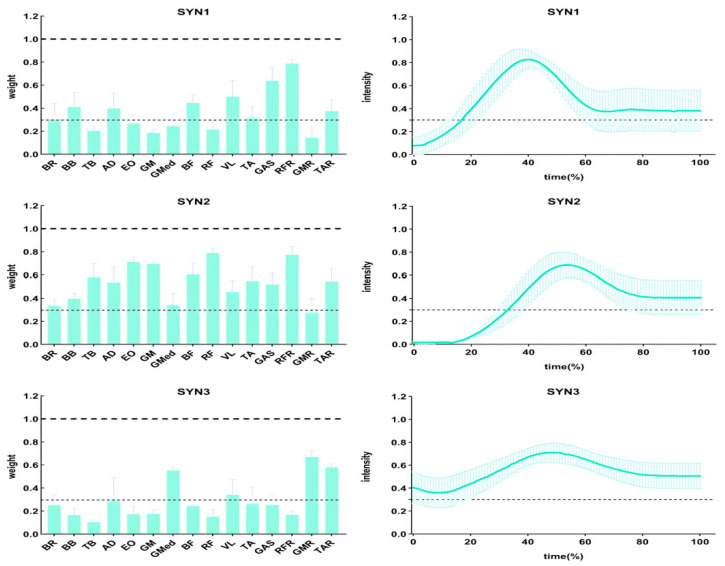
Muscle weights (**left**) and activation curves (**right**) for each synergistic muscle group of the SQG.

The muscle activation weights for the three extracted synergies (SYN1, SYN2, SYN3) across the ESG, RBG, and SQG protocols are presented in Table 4 and visualized in Figure 3, Figure 4 and Figure 5. Statistical analyses revealed that the spatial structure of the synergies was largely conserved, with significant between-protocol differences observed only in a limited number of specific muscles:

In SYN1, a one-way RM-ANOVA revealed a significant main effect of Intervention Protocol on the activation weight of the right rectus femoris (RFR) (F(2, 34) = 15.30, *p* < 0.001, ηp^2^ = 0.47). Post hoc comparisons indicated that the RFR activation weight was significantly higher in both the ESG and SQG groups compared to the RBG group (both *p* < 0.05).

In SYN2, significant main effects of Intervention Protocol were found for three muscles:

Gluteus maximus (GM) (F(2, 34) = 8.76, *p* = 0.001, ηp^2^ = 0.34): Post hoc tests showed the activation weight was significantly higher in the ESG group than in the RBG group (*p* < 0.05).

Rectus femoris (RF) (F(2, 34) = 12.18, *p* < 0.001, ηp^2^ = 0.42): Post hoc tests indicated the activation weight was significantly higher in both the ESG and SQG groups compared to the RBG group (both *p* < 0.05).

Vastus lateralis (VL) (F(2, 34) = 6.54, *p* = 0.004, ηp^2^ = 0.28): Post hoc tests revealed the activation weight was significantly higher in the RBG group than in the ESG group (*p* < 0.05).

In SYN3, a significant main effect of Intervention Protocol was found for the gluteus medius (GMED) activation weight (F(2, 34) = 18.45, *p* < 0.001, ηp^2^ = 0.52). Post hoc comparisons showed that the GMED activation weight in the RBG group was significantly higher than in both the ESG and SQG groups (both *p* < 0.05).

#### 3.2.2. Muscle Activation Coefficient

Table 5, Analysis of the temporal characteristics of synergy activation (activation duration, peak time, onset, and offset) indicated no statistically significant differences between the three intervention protocols for any of the synergies (all *p* > 0.05).

## 4. Discussion

### 4.1. Effects of Different PAPE Protocols on Vertical Jump Height and Mechanistic Analysis

This study compared the effects of three PAPE protocols on vertical jump performance among elite Sanda athletes, revealing distinct temporal and efficacy profiles. The findings are interpreted within the framework of neuromuscular activation mechanisms, load characteristics, and crucially, are contextualized by the methodology enabled by wearable sensor technology.

The primary finding was the superior and sustained performance of the Electrical Stimulation Group (ESG). At the 6-min post-intervention mark, ESG elicited significantly greater vertical jump heights compared to the Resistance Band Group (RBG) and Squat Group (SQG). This immediate potentiation suggests that the synergistic combination of mechanical loading and neuromuscular electrical stimulation (NMES) facilitates rapid optimization of neuromuscular pathways. This aligns with literature suggesting NMES can directly enhance motor unit recruitment efficiency, offering a temporal advantage over isolated mechanical loads [22]. The underlying mechanism may involve a dual activation: mechanical loading priming calcium regulatory processes, while electrical stimulation acutely elevates central nervous system excitability, collectively optimizing initial force production capacity [23].

A temporal divergence was observed at 8 min, where SQG reached its peak potentiation, significantly outperforming RBG but not differing from the maintained elevation of ESG. This pattern indicates that high-intensity mechanical loading (90% 1RM) induces a more delayed yet potent PAPE effect, consistent with known time-course characteristics of heavy resistance exercise. In contrast, the ESG protocol appeared to balance immediacy with persistence. By the 10-min assessment, only ESG continued to demonstrate a clear advantage over RBG. This prolonged effect in ESG may be attributed to the electrical stimulation component potentially extending the excitability of the neuromuscular system, thereby attenuating the early onset of fatigue-related inhibition [24].

Notably, the RBG protocol did not enhance vertical jump performance at any measured time point. This outcome may be attributed to two interrelated factors. First, the absolute load intensity (20% body weight) may have been insufficient to provide an adequate stimulus for the high-threshold motor units critical for explosive tasks. Second, and potentially more decisive, is a fundamental mismatch in movement specificity. The RBG intervention emphasized lateral resistance and hip abduction/stabilization. In contrast, both the vertical jump and the sport-specific side kick are dominated by sagittal-plane force production (hip and knee extension). This discrepancy in the primary direction and type of muscular effort likely resulted in minimal overlap in the neuromuscular pathways engaged by the conditioning activity and those required for the target performance, limiting any positive transfer [25].

### 4.2. Effects of Different PAPE Protocols on Lateral Kicking Muscle Synergy Patterns and the Role of Wearable Assessment

A central contribution of this study is the application of high-density, wireless surface electromyography (sEMG)—a paradigm of wearable sensing technology—to dissect the neuromuscular adaptations underpinning PAPE. This approach allowed for the non-invasive, ecologically valid capture of complex muscle coordination patterns during a dynamic sport-specific skill, moving beyond global performance metrics to examine the organization of the motor output itself [26].

#### 4.2.1. Spatial Reorganization of Muscle Synergies

Muscle synergy analysis via non-negative matrix factorization (NMF) revealed that different PAPE protocols induced distinct spatial adjustments in muscle activation weights, reflecting protocol-specific optimizations in neuromuscular control.

In the synergy primarily involving the right rectus femoris (SYN1), both ESG and SQG protocols were associated with a greater relative contribution from this key knee extensor compared to RBG. This suggests that high-intensity interventions, whether mechanical or combined, promote a heightened neural drive to prime movers [27].

More pronounced differences emerged in the synergy encompassing hip and knee extensors (SYN2). The ESG protocol was characterized by substantially greater contributions from the gluteus maximus and rectus femoris. A similar trend for rectus femoris was observed following SQG. This spatial shift towards greater activation of primary force-generators is biomechanically coherent with the demands of the kicking phase. Conversely, the RBG protocol showed a comparatively higher reliance on the vastus lateralis within this synergy [28]. This pattern may represent a compensatory strategy, where increased recruitment of a secondary knee extensor occurs when the drive to the primary hip and knee extensors is suboptimal, potentially reflecting a less efficient force-production strategy.

In a synergy associated with stabilization (SYN3), the RBG protocol demonstrated higher activation of the gluteus medius, consistent with the lateral stabilization demands of the band exercise. However, this isolated enhancement in a stabilizer muscle, decoupled from improved activation of prime movers, did not confer a performance advantage, underscoring the integrated nature of effective neuromuscular coordination [29].

#### 4.2.2. Temporal Stability of Muscle Activation

A key insight from the wearable sEMG data was the dissociation between spatial and temporal features of muscle synergies. While activation weights (spatial organization) showed clear inter-protocol differences, no significant changes were observed in the timing of synergy activations (onset, peak, offset, duration). This temporal stability indicates that in these elite athletes, the well-learned, automated sequence of the side kick was preserved [17]. The performance enhancement, therefore, appears to be mediated not by altering the timing of muscle coordination, but by refining the spatial recruitment strategy within the existing temporal framework. Specifically, the nervous system maintained the practiced coordination rhythm but increased the relative contribution of the most functionally relevant muscles during their allotted time windows, effectively increasing force output without disrupting movement architecture [30].

### 4.3. Research Significance, Practical Value, and the Future of Wearable-Driven Training

This study underscores the value of integrating wearable sensor technology, like wireless sEMG, into sports science research and practice. By providing a portable, high-fidelity window into the neuromuscular system during complex movements, such technologies enable a deeper, more objective analysis of intervention effects that goes beyond outcome measures.

Theoretically, the use of NMF on wearable sEMG data allowed us to demonstrate that PAPE effects are not monolithic but are expressed through specific, protocol-dependent adjustments in the spatial organization of muscle synergies. This enriches the mechanistic understanding of how pre-activation stimuli modulate motor control in skilled performers [31].

Practically, the findings suggest that the combined electrical stimulation protocol (ESG) may represent an efficient strategy for acutely enhancing performance in elite Sanda athletes. Its ability to rapidly and sustainably improve output, coupled with its optimization of prime mover recruitment, makes it a candidate for precision pre-competition priming. The failure of the RBG protocol highlights the critical importance of movement pattern specificity, a principle that may be even more vital for developing athletes [32].

Looking forward, the methodology employed here points toward a future of personalized, data-driven training. Wearable sensors can provide immediate, athlete-specific feedback on neuromuscular responses to different stimuli. This could guide the real-time selection and titration of warm-up or potentiation protocols based on an individual’s current physiological state and movement patterns, ultimately supporting more precise and effective training prescriptions in combat sports and beyond [33].

**Limitations:** This study has several limitations. First, while the analysis of six side-kick repetitions per condition is consistent with methodologies used for discrete, maximal-effort skills and yielded consistent synergy patterns (VAF > 90%), a larger number of trials could further enhance the robustness of muscle synergy extraction, particularly regarding the consistency of temporal activation profiles. Second, the muscle synergy analysis was confined to the spatial domain (activation weights); a formal comparative analysis of the temporal features of synergy activation between protocols was not conducted, which could provide a more complete understanding of neuromuscular adjustment. Third, the findings from this sample of high-level male Sanda athletes may not generalize to other populations (e.g., females, recreational athletes). Finally, the assessment was performed in a controlled laboratory setting, and the transfer of the observed PAPE effects to actual combat performance requires future verification.

## 5. Conclusions

This study compared the acute effects of three PAPE protocols on the neuromuscular performance of the side kick in elite Sanda athletes. The results indicate that the combined electrical stimulation protocol (ESG) was associated with the most favorable outcomes, characterized by immediate and sustained improvements in vertical jump height (from 6 to 10 min post-intervention) and concurrent, protocol-specific adjustments in muscle synergy patterns—notably an increased spatial contribution of key power-generating muscles (right rectus femoris, gluteus maximus, rectus femoris) during the movement. The traditional heavy squat protocol (SQG) produced a delayed peak effect on jump height at 8 min, with a shorter duration of benefit. In contrast, the resistance band protocol (RBG) did not enhance performance, which may be attributed to its lower load intensity and a mismatch between its lateral resistance vector and the sagittal-plane dominance of the kick.

Collectively, these findings suggest that the efficacy of a PAPE intervention is influenced by both the intensity of the stimulus and its congruence with the biomechanical demands of the target skill. The ESG protocol, which integrates these elements, appears to be a promising strategy for acutely potentiating lower-body explosive power and optimizing neuromuscular coordination relevant to the Sanda side kick, offering practical insights for pre-competition preparation.

## Figures and Tables

**Table 1:** Characteristics of subjects (n = 18).

Age (Years)	Height (cm)	Weight (kg)	Training Duration (Years)	Dominant Leg
22.5 ± 3.1	176.5 ± 6.2	70.2 ± 18.3	7.8 ± 2.5	Right

**Table 2:** Experimental Intervention Protocol.

Parameter	ESG (Electrical Stimulation + Squat)	RBG (Resistance Band)	SQG (Heavy Squat)
Primary Exercise	Barbell Back Squat	Band-Resisted Lateral Steps	Barbell Back Squat
Additional Stimulation	Synchronized Neuromuscular Electrical Stimulation (NMES) on quadriceps	None	None
Exercise Description	Squat synchronized with NMES	Band placed above knees; lateral stepping with controlled knee flexion (100–120°)	Squat with controlled depth (knee flexion < 90°)
Load Intensity	70% of 1RM	20% of Body Weight	90% of 1RM
NMES Parameters	80 Hz, 400 µs, 90% of maximal tolerated intensity	/	/
Volume	3 sets × 3 repetitions	3 sets × 15 m	3 sets × 3 repetitions
Inter-set Rest	3 min	2 min	3 min

ESG: Electrical Stimulation Group; RBG: Resistance Band Group; SQG: Squat Group.

**Table 3:** Comparison of Vertical Jump Height Between Groups at Different Time Points Following Three Interventions (cm, Mean ± SD).

Testing Time Point	SQG	RBG	ESG	F-Value/*p*-Value	Post Hoc Comparison (*p* < 0.05)
6 min after intervention	46.5 ± 3.2	47.0 ± 2.9	49.1 ± 2.8	F = 8.74, *p* = 0.002	ESG > SQG, ESG > RBG
8 min after the intervention	47.8 ± 3.1	46.2 ± 3.0	48.9 ± 2.7	F = 9.31, *p* = 0.001	ESG > RBG, SQG > RBG
10 min after the intervention	46.2 ± 3.3	45.7 ± 3.1	47.5 ± 2.9	F = 4.12, *p* = 0.032	ESG > RBG

ESG: Electrical Stimulation Group; RBG: Resistance Band Group; SQG: Squat Group.

**Table 4:** Weights for SYN Muscle Activation.

Muscle	Synergy	ESG	RBG	SQG
AD	SYN1	0.58 ± 0.23	0.36 ± 0.32	0.40 ± 0.30
BB		0.52 ± 0.19	0.48 ± 0.40	0.41 ± 0.28
BF		0.51 ± 0.21	0.46 ± 0.21	0.45 ± 0.16
BR		0.34 ± 0.30	0.49 ± 0.28	0.30 ± 0.31
EO		0.22 ± 0.14	0.34 ± 0.27	0.27 ± 0.17
GAS		0.42 ± 0.06	0.58 ± 0.44	0.64 ± 0.26
GM		0.09 ± 0.14	0.31 ± 0.29	0.19 ± 0.06
GMR		0.20 ± 0.08	0.36 ± 0.40	0.14 ± 0.21
GMed		0.21 ± 0.24	0.45 ± 0.14	0.25 ± 0.25
RF		0.22 ± 0.21	0.24 ± 0.17	0.21 ± 0.17
RFR		0.84 ± 0.06 *^a^	0.54 ± 0.07	0.79 ± 0.07 *^c^
TA		0.39 ± 0.23	0.46 ± 0.34	0.32 ± 0.20
TAR		0.35 ± 0.23	0.31 ± 0.28	0.37 ± 0.22
TB		0.32 ± 0.24	0.25 ± 0.10	0.20 ± 0.27
VL		0.35 ± 0.20	0.54 ± 0.32	0.50 ± 0.32
AD	SYN2	0.56 ± 0.07	0.46 ± 0.16	0.54 ± 0.36
BB		0.42 ± 0.22	0.39 ± 0.13	0.40 ± 0.13
BF		0.43 ± 0.16	0.61 ± 0.12	0.60 ± 0.27
BR		0.44 ± 0.17	0.50 ± 0.08	0.34 ± 0.15
EO		0.68 ± 0.17	0.67 ± 0.17	0.71 ± 0.12
GAS		0.48 ± 0.23	0.55 ± 0.22	0.52 ± 0.27
GM		0.81 ± 0.21 *^a^	0.53 ± 0.40	0.73 ± 0.14
GMR		0.38 ± 0.21	0.30 ± 0.28	0.28 ± 0.34
GMed		0.29 ± 0.29	0.47 ± 0.07	0.34 ± 0.29
RF		0.77 ± 0.208 *^a^	0.53 ± 0.23	0.78 ± 0.18 *^c^
RFR		0.81 ± 0.15	0.62 ± 0.27	0.79 ± 0.29
TA		0.50 ± 0.12	0.41 ± 0.34	0.55 ± 0.34
TAR		0.50 ± 0.23	0.51 ± 0.28	0.55 ± 0.31
TB		0.63 ± 0.20	0.52 ± 0.12	0.58 ± 0.34
VL		0.31 ± 0.30	0.54 ± 0.30 *^a^	0.43 ± 0.28
AD	SYN3	0.26 ± 0.26	0.32 ± 0.32	0.29 ± 0.41
BB		0.19 ± 0.20	0.21 ± 0.28	0.17 ± 0.11
BF		0.29 ± 0.14	0.41 ± 0.30	0.24 ± 0.08
BR		0.24 ± 0.16	0.23 ± 0.09	0.25 ± 0.18
EO		0.25 ± 0.23	0.32 ± 0.24	0.17 ± 0.14
GAS		0.24 ± 0.19	0.29 ± 0.30	0.25 ± 0.16
GM		0.29 ± 0.36	0.29 ± 0.27	0.18 ± 0.07
GMR		0.58 ± 0.32	0.63 ± 0.39	0.67 ± 0.10
GMed		0.56 ± 0.17	0.80 ± 0.14 *^a^	0.55 ± 0.04
RF		0.30 ± 0.47	0.29 ± 0.32	0.15 ± 0.12
RFR		0.27 ± 0.29	0.30 ± 0.31	0.17 ± 0.07
TA		0.40 ± 0.30	0.47 ± 0.32	0.26 ± 0.29
TAR		0.51 ± 0.39	0.62 ± 0.28	0.58 ± 0.06
TB		0.19 ± 0.20	0.09 ± 0.10	0.10 ± 0.04
VL		0.33 ± 0.43	0.46 ± 0.31	0.34 ± 0.26

Note: *^a^ indicates a significant difference between ESG and RBG; *^c^ indicates a significant difference between SQG and RBG. brachioradialis (BR), biceps brachii (BB), triceps brachii (TB), anterior deltoid (AD), external oblique (EO), gluteus maximus (GM), gluteus medius (GMed), biceps femoris (BF), rectus femoris (RF), vastus lateralis (VL), Tibialis Anterior (TA), Gastrocnemius Medial Head (GAS), as well as the right Tibialis Anterior (TAR), right Gluteus Maximus (GMR), and right Rectus Femoris (RFR).

**Table 5:** Coefficients for SYN Muscle Activation.

Synergy	Parameters	ESG	RBG	SQG
SYN1	Activation Duration T	166.33 ± 70.73	134.89 ± 74.23	192.40 ± 34.02
	Peak Moment T_max_	27.33 ± 7.09	32.44 ± 14.30	25.00 ± 4.69
	The Moment Begins T_start_	4.83 ± 8.54	6.67 ± 7.83	1.60 ± 3.58
	The End T_stop_	92.67 ± 17.96	88.89 ± 21.63	100.00 ± 0.00
SYN2	Activation Duration T	137.20 ± 24.16	118.14 ± 38.24	108.38 ± 44.91
	Peak Moment T_max_	48.60 ± 5.46	49.71 ± 7.32	48.88 ± 7.18
	The Moment Begins T_start_	30.00 ± 4.58	31.29 ± 7.45	30.38 ± 7.37
	The End T_stop_	89.40 ± 14.72	89.29 ± 13.60	86.00 ± 15.65
SYN3	Activation Duration T	197.29 ± 61.52	186.50 ± 64.61	155.64 ± 76.91
	Peak Moment T_max_	32.86 ± 28.07	34.50 ± 26.40	36.82 ± 28.69
	The Moment Begins T_start_	0.00 ± 0.00	2.88 ± 8.13	7.00 ± 12.33
	The End T_stop_	96.86 ± 8.32	97.25 ± 7.78	95.36 ± 10.43

## Data Availability

Informed consent was obtained from all participants involved in the study.
